# Epistemological implications of astroglia scientific research

**DOI:** 10.3389/fncel.2025.1718732

**Published:** 2025-11-28

**Authors:** Alfredo Pereira

**Affiliations:** 1 Universidade Estadual Paulista (UNESP), Institute of Biosciences, Botucatu-SP, São Paulo, Brazil; 2 Universidade Estadual Paulista (UNESP), Graduation Program in Philosophy, Marília-SP, São Paulo, Brazil; 3 Conselho Nacional de Desenvolvimento Científico e Tecnológico, Brasília, Brazil

**Keywords:** neuroglia, endogenous feedback, calcium wave, homeostasis, sentience

## Abstract

Neuroglia, comprising three cell types (astrocytes, oligodendrocytes and microglia), interact with neurons and extracellular components in brain physiology. Astroglia, having as main function the control of homeostasis, modulate dynamic processes in the nervous system, including mental functions; they are crucially involved in all neurological, psychiatric and degenerative disorders and diseases. How to change the century-old neuron-centered paradigm used to explain experimental phenomena in the clinical domain? This is the question addressed in this paper. I review a new explanatory paradigm based on an “endogenous feedback” between astroglial and neuronal networks: neuronal bioelectricity generates Local Field Potentials, which are synchronized, generating a dynamic field that impacts on a multi-ion population, releasing ‘shuttles’ that induce amplitude-modulated spatiotemporal patterns on astroglial ‘calcium waves’. The ‘calcium wave’ activates other signaling processes, as the release of ions in the “synaptic cradle,” to control the temporal dynamics of spike trains of the post-synaptic neuron and metabolic processes determining behavioral and endocrine responses. The “endogenous feedback” theoretical hypothesis can be tested by means of a combination of new techniques of visualization and analysis of amplitude-modulated spatiotemporal patterns present in astroglia *in vivo*, registers of behavioral patterns and subjective reports (in the case of alert persons under invasive brain surgery procedures), addressing the issue of how astroglial ‘calcium waves’ modulate neuronal dynamics, mediating brain processing of stimuli to produce adaptive responses.

## Introduction

Witnessing the richness of structures of living systems may be a delight for anatomists and morphologists, but often raises challenges for physiologists: what are the functions of all these cells, circuits and signals found in empirical investigations? This type of question is not absent in neuroglia research. Methodological and technological progress led to a cascade of discoveries of macro and micro structures, for which there is no well established function. The puzzle causes additional headache for biological investigations, since some of these functions may be not ordinary physical/chemical/biological functions, but related to *qualitative mental states* (as pain and pleasure) that cannot be felt by the scientist, but is experienced only by the observed system himself. In Philosophy, this reflexive operation is called the *first-person perspective* (Nagel, 1974), to be distinguished from the *third-person perspective* of the scientific observer, who is looking at the brain from the outside.

Qualitative mental states are the subjective sensations, feelings, emotions and meanings, accessible only to the person in whose brain the physiological processes addressed here occur. An hypothesis has been raised that the brain endogenous feedback (Carrara-Augustenborg and Pereira, 2012) provided by astroglia controlling brain homeostasis is at the biological roots of Sentience, our *capacity* for experiencing such qualitative states, understood as a necessary but not sufficient condition for consciousness (Pereira, 2021a, 2021b). In this case the studied processes are *psycho-physiological,* requiring special attention from neuroscientists to relate the physical components with the corresponding mental processes—an exercise that is often absent in the professional formation of neuroscientists.

The endogenous feedback between neuronal and astroglial networks is not taken into account in scientific explanations of brain physiology provided by the neuron-centered paradigm that began with the 1906 Medicine Nobelist Santiago Ramon y Cajal. His ‘Neuron Doctrine’ assign to neurons all functions of the brain, while glial networks were considered as a supportive tissue only. He gained more notoriety in the community of neuroscientists than the ‘syncytium’ view of Camilo Golgi, the second 1906 Nobelist. If the contrary notoriety happened, astroglia researchers would find more receptivity and application of their findings among contemporary neuroscientists.

The review below is divided in five sections:

- Problem and Hypothesis.
- From Neurons to Astrocytes.
- Dynamical Processes in the Astroglial Network.
- From Astrocytes to Neurons and Body.
- New Experimental Techniques and the Testing of the Hypothesis.

## Problem and hypothesis

A challenge for contemporary theoretical neuroscience is to approach both Mind/Brain and Neuron/Astroglia problems together, because they are closely related, in the following ways.

A very brief review of the computational neuroscience approach to physiology is convenient before presenting a hypothesis. The function of neuronal networks was elucidated by McCulloch and Pitts (1943), when they proposed a binary logical calculation representing the firing/non-firing of the neuron axon. This binary calculation is the basis of artificial machines that emulate biological cognition.

This approach was complemented in the 1980’s with the development of parallel architectures for the computer hardware, in which the pattern of neuronal connections was related to mental representations. Since then, there is no doubt among researchers that neuronal networks are related to information processing, performing functions close to human learning, memory and intelligence.

With the development of software that performs parallel operations using serial architectures, the concept that knowledge is based on patterns of connections of neuron-like units became popular, not only in the scientific community, but in several sectors of the “Information Society” we live in.

In the fascinating history of recent astroglia research, from 1990 to the present, the attempt to use the same neuron-centered methods and technologies to discover the functions of astroglia has not been so successful, possibly because their physiology is different. At this moment we enter a new, more complex heuristics, in which the investigation of biological functions of astroglia require a supplementary hypothesis about the connection between physiological and mental functions.

The physiology of neuroglia should be attached to their functions, which are different from neurons. If the function of neurons is to support information processing for a living system interacting with the physical and social environment, receiving information from several sensors and controlling behavior by means of muscles and glands, what could be the mental function of the astroglia that is ‘locked’ inside the nervous system, receiving informational input from some neurons and giving feedback to other neurons in the “astroglial cradle” (Verkhratsky and Nedergaard, 2014)?

In several publications (starting with Pereira and Furlan, 2009), I have addressed this issue with several co-authors, relating the mental function of astrocytes—considered to be the “Master Hub” for all neuroglia (Pereira and Furlan, 2010)—to *Sentience*, defined as “the capacity of feeling” (Pereira, 2021b), related to the information processed by neurons. Both cell types contribute to our mental functions.

Astroglia seems be involved in an “endogenous feedback” loop with neuronal networks, in which:

- The presynaptic neurons brings the information to be processed by the astroglial system,
- The astroglial system performs psycho-physiological (metabolic and motivational) functions different from the neuronal network (for a recent debate, see Bolaños et al., 2025), and.
- The neuroglial system feeds back to the post-synaptic neurons, controlling the timing of the spike trains they generate and send to muscles and glands.

The mental functions to be attributed to neuroglia are intimately connected to the physiological function of homeostatic control of the brain, proposed by Verkhratsky and Nedergaard (2018). This temporal process has been heuristically proposed to generate qualitative states (as sensation, perceptual ‘qualia’, feelings, emotions and meanings) for the person (Pereira, 2021a).

In the first-person perspective, mental life is dynamic and centered on a personal “here and now.” The quality of personal experiences are defined by what comes before and what is intended to come after. If we look for the neural correlates of such a “Flow of Consciousness,” the astroglial network, located between neuronal networks that process incoming information and neural networks that control action, appears as a perfect candidate to provide our capacity of “feeling what happens” (an expression used by Damásio, 1999) in our “here and now,” and, on the basis of this appreciation, to give an “endogenous feedback” to neurons that control our behavior.

## From neurons to astrocytes

In perceptual processes, sensory (‘bottom-up’) information reaches the central nervous system and elicits (together with internal ‘top down’ signaling; see Grossberg, 1999) the formation of Local Field Potentials (LFP). These dynamic fields spontaneously synchronize (Buszáki, 2006), activating the astroglial network simultaneously at several regions in the brain (Pereira and Furlan, 2010). The more activated regions increase blood flow, by an astroglial action on blood circulation. This distribution of activity appears in BOLD FMRI (in measurements with duration of around 2 s). Oscillatory synchrony was proposed to have the role of boosting communication between sensory neural LFP and astrocytes (Pereira and Furlan, 2009), inducing the amplitude-modulated patterns of the astroglial calcium waves that *respond* to the information content of multimodal perceptual scenes.

This process has three steps:

- Purinergic (Cotrina et al., 1998; Guthrie et al., 1999; Bowser and Khakh, 2007) and other signal transduction-controlled mechanisms impacting on calcium concentrations from the endoplasmic reticulum elicit the mechanical formation of ‘calcium waves’ *in single astrocytes*. The induction of a ‘calcium wave’ in one astrocyte requires an adequate level of presynaptic activity, integrating information from all input neurons. This assumption was modeled by Nadkarni and Jung (2003), concluding that their generation depends on the intensity of the input signal, which must be sufficient to raise calcium concentration above a threshold;
- The summation of the single astrocyte small waves by means of constructive interference through gap junctions generates a global calcium dynamics in the larger astroglial network, which is present in spatially separated and functionally distinct brain circuits. For this reason we raised the hypothesis that the astroglial network is the “Master Hub” of brain physiology (Pereira and Furlan, 2010);
- The global ‘calcium wave’ is frequency and amplitude modulated by the synchronous firing of neighbor neurons by mean of a multi-ion mechanism and related energy ‘shuttles’ (Bolaños et al., 2025; Andersen et al., 2025). In our psycho-physiological hypothesis of an “endogenous feedback” provided by astrocytes, the amplitude-modulated pattern of calcium dynamics has the motivational psychological function of giving a *valence* to the information processed by the neurons.

Findings by Schummers et al. (2008) showed that astrocytes in the visual cortex of ferrets are more sharply tuned to visual stimuli than neurons. Astrocytes’ responses arise from neuronal presynaptic activity, but the authors suggest that such an exquisite tuning is due to an existing threshold level that must be crossed to elicit ‘calcium waves’. Such a level would be reached only with neuronal activity coordination. In fact, when synaptic transmission was only slightly decreased, a significant fall in the astrocytes’ responses was observed.

The above findings gave empirical support to mathematical and computational models of the generation of ‘calcium waves’ in one astrocyte participating in an ensemble of synapses. Further hypotheses about the threshold for induction of ‘calcium waves’ in astrocytes were formulated by De Pittà et al. (2008). This model covers the process by which external (e.g., sensory) signals modulate IP3 activity. The summation process that results from synchronized multiple inputs induces both amplitude and frequency-modulated ‘calcium waves’. In neurons, spike trains are modulated only by frequency; amplitude modulation of calcium dynamics in astrocytes is probably related to their proper function.

Local synchrony in several frequencies—depending on the properties of specialized circuits, e.g., theta frequency in the hippocampus—can boost neuro-astroglial communication in each brain region (Pereira et al., 2012). Dominant low frequency waves, as in slow wave sleep, are related to lower amplitude calcium dynamics, and dominant higher frequencies and amplitudes are related to arousal. Most of the evidence about the correlation of high-frequency oscillatory synchrony and conscious states was obtained with recordings of gamma activity. A framework has been proposed that focuses on phase locking of gamma with alpha and beta rhythms (Palva and Palva, 2007). A dynamical picture can be formed, where gamma is considered as being related to the stabilization of attentional focus on perceptual patterns, while alpha rhythms mediate transitions between different patterns. On the basis of these findings, it is possible to construct a theoretical view of the combination of brain rhythms with astroglial network frequencies (see Pereira et al., 2012).

In mnemonic processes, it is also possible that theta rhythms in the medial temporal lobe are phase-locked with the higher frequency rhythms found in other regions of neocortex and eventually coordinate them. The frequency of coordinated spiking determines the amount of glutamate release from neurons to astrocytes; in Gamma, the amount of glutamatergic release reaches the threshold necessary to produce coherent ‘calcium waves’ in astrocytes, while in delta, this level of activity remains at subthreshold levels.

In summary, the generation of astroglial ‘calcium waves’ by the surrounding neuronal population requires multiple coordinated inputs that reach an excitation threshold. Synchronization of neuronal activity (including graded and action potentials, i.e., oscillatory synchrony and spike synchrony) can enhance communication through phase coordination of excitatory inputs; therefore, global synchrony is likely to facilitate the transfer of cognitive patterns embedded in neuronal local field potentials (LFPs) to astroglial calcium waves, where they elicit a *motivational* response.

## Processes in the Astroglial network

Phase locked higher-frequency rhythms operate together to boost neuro-astroglial communication, transferring LFP patterns to astroglial activity, where these patterns are integrated, forming amplitude-modulated temporal patterns relevant to brain homeostasis. The endogenously generated reaction of the brain to the perturbation determines the responses to the stimuli that generated LFPs. This cycle composes the “endogenous feedback,” in which neurons excited by incoming stimulation activate the adjacent astrocytes, causing the formation of amplitude-modulated patterns in astroglial ‘calcium waves’. We have estimated that this process occurs in temporal windows of around 2 s (Pereira et al., 2017).

Neuronal assemblies displaying oscillatory synchrony at different frequencies (Palva and Palva, 2007) contribute to the formation of 2-s astroglial waves in a cooperative way. Pereira et al. suggest that:

“The 2-s interval corresponds to a whole brain cycle of 0.5 Hz. Nested in this cycle, we find the Slow Cortical Potential (SCP) with 1 Hz and the nested EEG waves with different frequencies. The 2-s waveform, corresponding to the whole cycle of the calcium wave, contains two cycles of the SCP. Buszáki (2006, p. 354, footnote 41) analogizes the ratios with respect to the Fibonacci series. To illustrate this kind of mathematical pattern, the progression of frequencies during the 2-s time when a conscious episode is formed can be covered by the recursive equation Y = 2X + 0.5. Beginning with a frequency of 1 Hz (corresponding to the SCP.), we have for the first six recursions the series: 2.5; 5.5; 11.5; 23.5; 47.5 and 95.5 Hz, which roughly correspond to brain rhythms involved in conscious processing. This kind of approach was anticipated by Kozma and Freeman (2003) and Buszáki (2006, p. 369–370...), who have suggested that a recursive operation underlies the brain self-organizing oscillatory activity” (Pereira et al., 2017, p. 9).

Such calcium waves can be driven by distinct mediators as acetylcholine, GABA, ATP and glutamate (see review in Pereira and Furlan, 2010). The active receptors raise, among other signaling pathways, intracellular levels of phospholipase-dependent inositol triphospate (IP3), which release calcium from internal stores. The calcium dynamics of the astroglial network can be directly visualized by means of two-photon microscopy and more recent techniques, or indirectly assessed by the BOLD FMRI signal. Upon neuronal activation, in spite of the expected elevation in deoxygenated blood level, there is an increase in local cerebral blood flow. This increase occurs in such a degree that the delivery of oxygenated blood surpasses oxygen consumption in those more active areas. As a consequence, the BOLD signal is enhanced (Logothetis et al., 2001). Astrocytes have a critical role of in such processes (see, e.g., Zonta et al., 2003); they release vasoactive substances such as nitric oxide and metabolites of arachidonic acid that modulate neurovascular coupling. The BOLD signal is, therefore, indirectly related to astroglial activity, since astrocytes drive hemodynamic changes that supply energetic demands to active brain areas.

When chemically or physically stimulated, astrocytes do not generate the typical nonlinear membrane electrical changes observed in neurons, but show oscillations in cytosolic calcium levels as response. The amplitude of ionic vibrational energy changes in time and space, appearing in videos produced with two-photon microscopy (e.g., in the supplementary material provided by Resendez et al., 2016). The ‘calcium wave’ is experimentally visualized as changes in intensity of light in two-photon microscopy, because the changes of energy cause release and absorption of photons. The amplitude of the wave increases in the sleep-waking transition; also lactate release levels increase, as studied by Lundgaard et al. (2017); the goal of that study was to understand sleep and glympathic clearance, but they also found that lactate concentrations are higher in the waking state. In microarousals, there is also a fluctuation of noradrenaline levels (Lüthi and Nedergaard, 2025), suggesting a combined mechanism of energy supply and neuromodulation in the sleep-arousal dynamics.

What is the biophysical phenomenon behind the ‘calcium wave’ visualized with two-photon microscopy? This technique uses photons to access the calcium dynamics; the physics behind this process can be understood in terms of Quantum Electrodynamics (Feynman, 1985) and Ion-Trap computing (Pereira and Polli, 2006). A brief explanation goes like this: When ions receive external kinetic energy, its resulting vibrational state (“phonon”) changes; at this moment, the ion emits photons that reaches the next ion (and the microscope), causing the same type of change, affecting the next ion in the chain, and so on. Therefore, in the ‘calcium wave’ phenomenon, the ions are not moving in space (it is not a ‘travelling wave’ as those of water in the ocean, in which the molecules are displaced), but there is a wave of kinetic energy (see Mentré, 2012). This phenomenon was called “the domino effect”) (Pereira and Furlan, 2010).

Our proposal (Pereira and Polli, 2006) was initially based on the Los Alamos quantum computing model using Ca+. We received the objection that biological calcium is not Ca+, but Ca++. To overcome the problem, the concept of “The Hydro-Ionic Wave” was developed (Pereira, 2017), in which vibrating cations (Ca++, K+ and Na+) repel each other, forming the wave. There is also a proposed extracellular function for H protons (Pereira, 2020). In this case, the action of electromagnetic fields (LFPs) would impact on the multi-ion population, generating the amplitude-modulation of the astroglial calcium dynamics.

This sequence of events in the living brain can be summarized as: Neuronal bioelectricity generates LFPs; LFPs are spontaneously synchronized (Buszáki, 2006), generating a dynamic field; this dynamic field moves ions at several locations in the brain, generating electromagnetic patterns that modulate the amplitude of the existing astroglial calcium wave; the global calcium dynamics modulates neuronal activity at all active regions in the brain (see Fellin et al., 2004); this modulation was called “carousel effect” (Pereira and Furlan, 2010). The whole 2-s cycle composes the abovementioned “endogenous feedback,” which putatively carries mental functions.

Biophysical models of calcium dynamics in intracellular domains predict a complexity of amplitude- and frequency-modulated wave patterns. According to Musotto et al. (2025):

“The aim of the mathematical models analyzed in this contribution is to interpret the emergence of complex intracellular Calcium dynamics as the result of interdependent Ca^2+^ fluxes between the cytosol and intracellular stores, driven by the interaction with IP_3_. The models are described by systems of non-linear ordinary differential equations (ODEs), which are capable of supporting self-sustained Calcium oscillations. These phenomenological models have been developed to reproduce Calcium flow behavior comparable with available experimental data and have played a crucial role in the advancement of neuroscience, serving as a bridge between experimental observations and the development of more in-depth theories” (Musotto et al., 2025, p. 11–12).

In a broad approach, the hypothesis can be raised that four ‘biological’ ions—as well as protons—are involved in shaping the amplitude of astroglial calcium dynamics, composing a multi-ion wave (Pereira, 2017; Verkhratsky et al., 2020). According to the conjecture, the amplitude-modulated patterns of the astroglial calcium wave are shaped by the patterns of stimuli, together with previously existing patterns from long- and short-term memories (Grossberg, 1999). The above Fibonacci-like combination of frequencies composes the multiplex carrier wave of calcium dynamics, while the waveforms defined by *temporal amplitude modulation* of the carrier wave would correspond to the ‘valence’ of the wave, putatively related to the motivational qualitative experience that the owner of the brain is experiencing.

At this point, the neuron/astroglia problem merges with the mind/brain problem; the proposed hypotheses are intended to provide a common solution for both: The formation of the astroglial ‘calcium wave’ corresponds to the adaptive reaction of the living organism to the sensory/perceptual scene; this reaction drives the internal homeostatic response, providing an evaluation of the valence of the perceptual scene, and also modulates the firing of the neurons that control muscular and endocrine actions. Together with the generation of the response, the agent subjectively experiences the qualities of the patterns related to these metabolic processes (Pereira, 2011a, 2011b).

## From astrocytes to neurons and body

The results of astroglial network processing define the feedback signal to synapses and to target cells located in the “astroglial cradle” (Verkhratsky and Nedergaard, 2014) and connected to encephalic microcirculation. Several gliotransmitters, molecules and ions, released by astrocytes, interact with receptors located at both pre- and postsynaptic neurons, including glutamate, D-serine, Sodium, Potassium, Protons and ATP. This feedback has consistently been related to the control of whole brain homeostasis (Verkhratsky and Nedergaard, 2018). The ‘valence’ of the homeostatic response to stimuli perturbations modulates neuronal firing to muscles and glands, vascular control (vasodilation or constriction), and also diffusion of chemical signals through extracellular fluid ramifications in the body.

There is a causal relation between the dynamics of calcium waves in astrocytes and the corresponding rise of calcium entry in adjacent neurons (Pasti et al., 1997). The first publications suggesting this relationship were published 30 years ago, and a number of those experiments indicated that the mechanism underlying such a response include glutamate release from astrocytes and the consequent activation of ionotropic glutamatergic receptors at neuronal locations, as well as the release of potassium ions, modulating—with oligodencrocytes—the timing of spike trains along the neuronal axon (Charles, 1994; Parpura et al., 1994; Hassinger et al., 1995).

Activation of extrasynaptic neuronal NR1/NR2B NMDAR by the astroglial ‘calcium wave’ reaching the synaptic cradle contributes as an ‘endogenous feedback’ to reinforce synchronized neuronal assemblies (Fellin et al., 2004; Angulo et al., 2004). Slow calcium currents from the cradle to NR1/NR2B NMDAR have higher amplitude and slower kinetics than the influx currents mediated by NR1/NR2A, showing larger rise and decay times (Haydon and Carmignoto, 2006; Parri et al., 2001). More recent results show that sodium signaling is also present in homeostatic cascades involving slow calcium currents in extrasynaptic NMDA receptors (Untiet et al., 2025), whereas Chloride is involved in regulation of inhibition (Untiet et al., 2023). A recent review covers several possibilities in this area of research (Imrie and Farhy-Tselnicker, 2025).

In regard to the feedback action of astrocytes on neurons providing brain homeostasis, there are many transporters (including K + shuttling) taking part in feedback/forward communication between neurons and astrocytes, with distinct functional roles. Based on experimental studies Verkhratsky and Rose (2024) claim that “astrocytic excitability is based on cytosolic signals generated not only by Ca^2+^ but also by Na^+^ and Cl^−^ and H^+^ (and possibly even by K^+^, which remains to be investigated). Moreover, Na^+^ signaling is fundamental for real time coordination between neuronal activity and astroglial homeostatic support, which is critical for normal CNS function.”

In regard to energy metabolism, there is a current controversy on the substrate for activation of neurons at the astroglial cradle. Some authors propose a central role of the “lactate shuttle” (see references in Bolaños et al., 2025), while others stress the diversity of “shuttles” at the cradle (Andersen et al., 2025), or propose a more proeminent role for glycolitic upregulation (Dienel et al., 2025). From our previous conjectures, shuttles in the astroglial cradle are likely to boost synchronization of neuron oscillations (using Na + and K+) in faster frequencies (theta and above), and then neuronal synchrony boost the increase of amplitude of Ca^2+^ oscillations in astrocytes. It is a positive feedback loop until inhibitory neurons (using Cl–) are activated and then it becomes a negative loop.

A new pathway for an astrocyte network-whole body communication has recently been discovered (Li et al., 2025), having cerebrospinal fluid as a medium for signaling from the synaptic cradle and spinal nerves to peripheral organs. According to Novakovic and Prakriya (2025), “The astrocyte Ca^2+^ signaling toolkit consists of many components producing distinct Ca^2+^ signatures. Astrocyte Ca^2+^ signaling drives inflammation through gene expression, gliotransmission, and metabolism.” If well confirmed, these findings should be relevant for alternative medicine, including the placebo effect, healing meditation, acupuncture, and psychosomatic effects in general.

## New experimental techniques and testing of the hypothesis

The detection of waves *in vivo* requires the opening of the skull of brainy animal, posing an obstacle for human studies. In other animal species used in the Lab, several techniques have been developed, as genetically encoded calcium indicators for photon imaging, fiber photometry, light-sheet microscopy, and random-access multiphoton microscopy, and the use of acousto-optics deflectors (Salomé et al., 2006) to improve the resolution of two-photon microscopy.

These techniques allow better analysis of the measured dynamic patterns to test our theoretical hypothesis. The “endogenous feedback” hypothesis can be tested by means of a combination of:

1. New techniques of visualization and analysis of amplitude-modulated spatiotemporal patterns present in astroglia *in vivo*,
2. Registers of behavioral patterns and.
3. Subjective reports (in the case of alert persons under invasive brain surgery procedures), addressing the issue of how astroglial ‘calcium waves’ modulate neuronal dynamics, mediating brain processing of stimuli to produce adaptive responses.

For instance, fiber photometry allows the identification of brain-mind-behavior correspondences *in vivo,* using fluorescent molecules, including dynamic patterns involved in mental processes such as motivation (Simpson et al., 2024). Advances in two-photon microscopy combined with fluorescent tagging of calcium receptors has allowed important discoveries about the concentration of calcium in vivo (Resendez et al., 2016) that confirm that changes in waking states are accompained by changes in amplitude modulation of the astroglial calcium wave.

## Concluding remarks

In this paper I summarized a hypothesis about the double, psycho and physical function of the astroglial network: the control of homeostasis, and generation of mental states that confer a ‘valence’ to the information being processed in neuronal networks. Considering this role in the context of the whole living brain, it supports the broader hypothesis of an ‘endogenous feedback’ between astroglial and neuronal networks (the ‘carousel effect’ in Pereira and Furlan, 2010; conceptually developed in Carrara-Augustenborg and Pereira, 2012).

These theoretical hypothesis allow a reconfiguration of three key concepts at the foundations of Brain Sciences: Homeostasis, Feedback and Signal Transduction (in other words, embodied information processing generating meaning for the person owner of the brain). If accepted, they can inspire a healthy epistemological paradigm change, considering neuroglia as equal partners of neurons in the generation of brain and mental ‘covert’ and ‘overt’ functions.

## Data Availability

The original contributions presented in the study are included in the article/supplementary material, further inquiries can be directed to the corresponding author/s.
